# Psychological adjustment and growth of graduate nursing students under the dual stress of research and practicum: a qualitative study

**DOI:** 10.3389/fmed.2026.1734998

**Published:** 2026-05-29

**Authors:** Ruoxuan An, Jing Cheng, Xuefen Lan, Xiaotong Han, Wancheng Liao, Liandan Lin, Minhua Chen, Yanni Zhang

**Affiliations:** 1 Lishui People’s Hospital (The First Affiliated Hospital of Lishui University), Lishui, Zhejiang, China; 2 The First Affiliated Hospital of Guangzhou Medical University, Guangzhou, China

**Keywords:** academic stress, clinical practice, graduate nursing students, psychological adaptation, resilience, thematic analysis

## Abstract

**Background:**

As China’s nursing career develops and the demand for nursing positions in hospitals changes, the number of people applying for a master’s degree in nursing has been increasing year by year.

**Objective:**

To explore the psychological experiences of graduate nursing students under the dual pressures of clinical nursing practice and academic research and the factors that influence them.

**Method:**

Fifteen participants were recruited from 11 universities in China between October and December 2024 using a purposive sampling method. Semi-structured in-depth interviews were conducted and transcribed verbatim. Inductive reflexive thematic analysis was conducted using Braun and Clarke’s framework.

**Result:**

Four themes were identified: negative experiences of dual stress, coping mechanisms for negative experiences, positive effects of dual stress, and the inner need for graduate nursing education. Nursing students improve their general competence through internships and research studies and have a clear understanding of their inner needs and plans. However, students also have adverse psychological experiences at this stage for various reasons. As a result, they use a variety of methods to solve their problems, aiming to face their internships and research work in a better state and complete their graduate studies.

**Conclusion:**

Schools and hospitals should pay more attention to the needs of nursing students under dual stress, monitor their emotional state, and provide support in all areas to enhance their coping skills.

## 1 Introduction

With the continuous development of nursing education in China, nursing educators and administrators are paying more and more attention to the training of postgraduate nursing students (1, 2). Hospitals require nurses not only to focus on clinical work, but also require them to have a certain degree of scientific research ability to carry out academic research, to achieve a combination of scientific research and practice, in order to promote the development of the nursing discipline (3, 4). The number of postgraduate nursing graduates in China has increased significantly in recent years, and the proportion of nursing graduates with a master’s degree is on the rise (5). The Master of Science in Nursing is the future of healthcare as the new force of the nursing profession. Postgraduate nursing students will move into clinical practice after completing their theoretical studies in school. Clinical practice is a critical period for postgraduate nursing students to accumulate and expand their clinical knowledge and skills, establish positive professional values, and lay the foundation for good professional behaviors and literacy (6, 7). Unlike other nursing interns, postgraduate nursing students are often physically and mentally exhausted as they are not only faced with heavy clinical work, but also with difficult research tasks and studies (8–10) (this process is called the “dual stress period” between practice and study). Therefore, the period of dual pressure refers to the convergence of the internship and postgraduate stages. If they cannot adapt to the environmental changes during this period and deal with the negative experiences correctly, they are prone to produce a series of negative psychology (11), resistance to internship and academic research, and reduce the sense of professional identity (12), which not only directly affects the quality of clinical nursing teaching, but also has an adverse impact on the development of nursing talent team (10). Therefore, it is crucial to understand the psychological experiences of trainee postgraduate nursing students under dual stress. This provides important clues for maintaining the mental health of postgraduate nursing students, improving the management and training system of postgraduate nursing students and promoting the development of the profession (13). However, there is a lack of research on the psychological state of postgraduate nursing students under dual stress and the development of targeted intervention programs. The aim of this study is to explore their psychological experience and its influencing factors in depth, so as to provide reference and guidance for constructing clinical nursing education and psychological interventions for postgraduate nursing students.

## 2 Methods

### 2.1 Design

This qualitative descriptive study used online interviews to collect qualitative data and analyze it thematically. It followed the guidelines of the Comprehensive Standards for Reporting Qualitative Research (14) (Table 1).

**Table 1:** Consolidated criteria for reporting qualitative research guidelines.

No. item	Guide questions/description
Domain 1: Research team and reflexivity	
1. Interviewer/facilitator	Which author/s conducted the interview or focus group?
2. Credentials	What were the researcher’s credentials? e.g., PhD, MD
3. Occupation	What was their occupation at the time of the study?
4. Gender	Was the researcher male or female?
5. Experience and training	What experience or training did the researcher have?
6. Relationship established	Was a relationship established prior to study commencement?
7. Participant knowledge of the interviewer	What did the participants know about the researcher?
8. Interviewer characteristics	What characteristics were reported about the interviewer/ facilitator?
Domain 2: Study design	
9. Methodological orientation	What methodological orientation was stated to underpin the study? e.g., content analysis
10. Sampling	How were participants selected?
11. Method of approach	How were participants approached?
12. Sample size	How many participants were in the study?
13. Non-participation	How many people refused to participate or dropped out? Reasons?
14. Setting of data collection	Where was the data collected?
15. Presence of non-participants	Was anyone else present besides the participants and researchers?
16. Description of sample	What are the important characteristics of the sample?
17. Interview guide	Did the authors provide questions, prompts, and guides? Was it pilo*t*-tested?
18. Repeat interviews	Were repeat interviews carried out? If yes, how many?
19. Audio/ visual recording	Did the research use audio or visual recording to collect the data?
20. Field notes	Were field notes made during and/ or after the interview or focus group?
21. Duration	What was the duration of the interviews or focus groups?
22. Data saturation	Was data saturation discussed?
23. Transcripts returned	Were transcripts returned to participants for comment and/ or correction?
Domain 3: Analysis and findings	
24. Number of data coders	How many data coders coded the data?
25. Description of the coding tree	Did the authors describe the coding tree?
26. Derivation of themes	Were themes identified in advance or derived from the data?
27. Software	What software, if applicable, was used to manage the data?
28. Participant checking	Did participants provide feedback on the findings?
29. Quotations presented	Were participant quotations presented to illustrate the themes/findings? Was each quotation identified?
30. Data and findings consistent	Was there consistency between the data presented and the findings?
31. Clarity of major themes	Were major themes clearly presented in the findings?
32. Clarity of minor themes	Is there a description of diverse cases or a discussion of minor themes?

### 2.2 Participants

Participants were recruited from October 2024 to December 2024 from eleven universities with master’s degree programs in nursing in the east, west, south, and north of China. Eligible participants included graduate nursing students currently experiencing ‘dual stress’ situations, students on leave of absence for less than a week, and individuals willing to sign an informed consent form. In this study, a total of 15 respondents were selected through purposive sampling, and none withdrew. Participant information is shown in Table 2. After informed consent was obtained, an online videoconference interview was arranged.

**Table 2:** Characteristics of the study participants (*n* = 15).

Number	Age	Gender	Education background	University	Trainee hospital (grade)	Internship duration (month)
N1	25	Female	Full-time graduate student	Lishui University	Lishui People’s Hospital (Grade III Level A)	8
N2	23	Female	Full-time graduate student	Shanghai Jiaotong University	Ruijin Hospital (Grade III Level A)	9
N3	23	Female	Full-time graduate student	China Medical University	Affiliated Hospital (Grade III Level A)	6
N4	25	Female	Full-time graduate student	Shandong University	Affiliated Hospital (Grade III Level A)	6
N5	24	Female	Full-time graduate student	Hunan Medical University	Xiangya Hospital (Grade III Level A)	10
N6	23	Female	Full-time graduate student	Jinan University	Guangzhou Hospital (Grade III Level A)	7
N7	25	Female	Full-time graduate student	Shantou University	Affiliated Hospital (Grade III Level A)	9
N8	23	Female	Full-time graduate student	Zhejiang Medical University	Yanpu Community Hospital (Grade III Level A)	7
N9	25	Female	Full-time graduate student	Nanchang University	Affiliated Hospital (Grade III Level A)	9
N10	25	Female	Full-time graduate student	Shandong Medical University	Shandong Provincial Hospital (Grade III Level A)	5
N11	23	Female	Full-time graduate student	Guangxi Medical University	Affiliated Cancer Hospital (Grade III Level A)	11
N12	26	Male	Full-time graduate student	Guangxi Medical University	Affiliated Cancer Hospital (Grade III Level A)	11
N13	25	Male	Full-time graduate student	Guangxi Medical University	Affiliated Cancer Hospital (Grade III Level A)	11
N14	24	Male	Full-time graduate student	Lishui University	Lishui People’s Hospital (Grade III Level A)	9
N15	26	Male	Full-time graduate student	Henan University	Nanyang Central Hospital (Grade III Level A)	15

In China, postgraduate nursing students usually start their clinical practice after completing their first year (including the summer and winter holidays after the first year). Therefore, the dual -pressure period refers to the intersection of internship and graduate school.

### 2.3 Data collection

After reviewing the literature and considering the study’s objectives, the interview outline was initially designed and refined through a pilot interview with a postgraduate nursing student under dual stress.

Includes final interview questions:

(1) What negative experiences have you had with clinical nursing placements and nursing research?
(2) What were the reasons that brought you to these experiences?
(3) How have these experiences affected you?
(4) How do you alleviate the dual stress of your placement and research?
(5) What are your needs above research and clinical placement?

The first author was the interviewer for this study, a postgraduate nursing student trained in systematic qualitative research methods who was able to complete the interviews independently and successfully. In each interview, the interviewer was assigned to conduct a semi-structured, in-depth interview with the respondent (15). Prior to the style interview, the researcher contacted the participants via messaging software to negotiate the time of the interview to ensure that neither the interviewer nor the participant would be disturbed by external factors during the interview. The researchers then logged into Tencent Meetings (a feature-rich videoconferencing software with clear video and audio quality, similar to Zoom.) and scheduled meetings for specific time slots. Informed consent was obtained within half an hour prior to the start of the interview when the researcher sent the link to the scheduled session to the participant via messaging software. During the interview, the interviewer uses simple language, avoids ambiguous statements, maintains a smiling demeanor, shows respect for the participant, and provides appropriate responses (e.g., nodding or other encouraging gestures).

The study strictly adhered to the principle of informed consent, protected the privacy of the respondents, and audio-recorded with their permission. Because it was an online interview, online consent was used. The specific approach was to send the study’s informed consent form to each respondent for reading, inform them of the precautions and recording situation, and after obtaining permission, the respondent could reply one-on-one in the chat software interface to agree to proceed. The interviewer maintained an objective and neutral stance throughout, listened attentively and observed the facial expressions and body language of the interviewees, which were also noted. Non-verbal expressions were primarily understood through body language, such as the respondent’s tone, facial expressions, and movements, based on certain psychological research methods, repeated listening to recordings, and verified with the respondents, to avoid the author’s subjective bias. The text analysis and transcription were conducted by two nursing postgraduates who had undergone systematic research training, each analyzed and coded independently, and finally summarized and compared, revisited the original data to eliminate doubts. Experts, such as senior qualitative researchers, were also consulted to provide judgment and insights.

The interviewer made a brief note of the interview at the end of the interview. The interviews lasted 30–50 min, and the audio recordings were transcribed into text within 24 h of the interviews. Each participant was interviewed only once, which avoided repeat interviews. Data collection reached saturation after 12 interviews as no new codes or themes emerged (15). To further confirm that there were no new themes, three additional interviews were conducted. Data collection stopped after the 15th interview to ensure that no new themes emerged (16).

### 2.4 Data analysis

The study used the thematic analysis method proposed by Braun and Clarke (17) for data analysis. Organize synthetic data using NVIVO 12.0. The thematic analysis approach proposed by Braun and Clarke consists of six steps: familiarizing oneself with the data, generating an initial code, searching for themes, reviewing the themes, defining and naming the themes, and writing the manuscript.

The researchers watched the interview videos repeatedly, scrutinizing the respondents’ tone of voice, facial expressions, and body language to ensure the accuracy and reliability of the data. When different participants reported the same fact but with different tones and body language, this indicated that they held different attitudes toward that fact—a phenomenon that was analyzed in depth. When participants held a positive view of an experience, they expressed excitement and shared it enthusiastically with the interviewer. Conversely, when they held a negative view of an experience, they slowed their speech, accompanied by a range of body language, such as a bitter smile, a tilt of the head, or a shrug. Therefore, the interviewer briefly recorded changes in the rhythm of the respondent’s tone of voice and body movements during the interview in order to better identify these cues when reviewing the video. By interpreting the tone of voice and body language, the researchers were able to understand what the respondent meant.

In this study, the data were tree-coded based on the interviews, and a total of 155 codes were generated during the initial coding process, including 661 reference points (to familiarize with the data and generate initial codes). The initial codes were then integrated into the potential categories to form 48 new codes. A further 48 codes were integrated to find themes and sub-themes (search themes). Review videos of interviews and improve analytics to ensure proper alignment between themes and code and between different themes (audit themes). Discuss the generated codes with the research team and nursing specialists under their guidance until a consensus is reached. After optimizing the themes, the researcher will provide appropriate descriptions and names for each theme based on the theme’s focus and coding type to ensure that they are concise and comprehensive (Defining and Naming Themes). Eventually, four themes with 2–3 sub-themes each were developed for this study, and the results were made available to the respondents.

### 2.5 Rigor

Credibility, confirmability, and reproducibility were used as criteria to assess the rigor of this study (18). The researcher establishes a good trusting relationship with the participants prior to the interviews and expresses empathy during the interviews to ensure the appropriateness and correctness of the data, thus ensuring the credibility of the study. The findings represent data collected without subjective bias, and the researchers were trained in systematic qualitative research methods to ensure confirmability and reliability. The transferability of the study was ensured by the detailed description of the data and the maintenance of a reflective record. Participants had a clear program of postgraduate study, and all had placements in tertiary hospitals to ensure the data was collected in a representative manner.

During the interviews, the researcher listened attentively, did not interrupt the participants, and maintained a neutral stance. Participants were encouraged to express their inner feelings and ensure that their personal information was kept confidential. The researchers further investigated the participants’ responses for more detailed information. Non-verbal communication was used to express empathy and understanding of the participants’ experiences. Video and audio of the interviews were retained to record the participant’s body language and tone of voice changes. The researchers completed the transcription within 24 h of each interview and then verified it with the participants to ensure accuracy.

### 2.6 Ethical considerations

The study was approved by the Ethics Committee of Lishui University School of Medicine (2024YR 047). The written informed consent for participating in this study is provided by the participants themselves. In addition, written informed consent has been obtained from the participants for the publication of any potentially identifiable images or data included in this article. Following the principle of confidentiality, participants had the right to withdraw from the study at any time and without reason.

## 3 Results

Fifteen participants (Table 2), including three males and 12 females aged between 20 and 27 years, took part in the study. Ultimately, four main themes and 11 sub-themes were identified (Table 3).

**Table 3:** Main themes and sub-themes.

Main themes	Sub-themes
Negative experiences of “double pressure.”	Reduced enthusiasm for research
	Lack of learning resources
	Difficulties in clinical practice
Coping mechanisms of negative experiences	internal regulation
	External support
Positive effects under double pressure	Tougher willpower
	Strengthening of research capacity
	Improvement of overall quality
Inner need	Adequate research resources
	Flexible internship management model
	Granting of financial assistance

The four main themes were negative experiences of “dual stress,” coping mechanisms for negative experiences, positive effects of dual stress, and the need for graduate nursing education.

### 3.1 Negative experiences of “dual pressure”

Participants described negative experiences that arose during their internships and studies, which affected their productivity, enthusiasm for research, and even their lives. Over time, these adverse experiences have become a significant contributor to the decline of their professional identity in the nursing field.

#### 3.1.1 Reduced enthusiasm for research

##### 3.1.1.1 Peer pressure and competition

The phenomenon of peer pressure refers to the psychological stress that individuals in the graduate nursing student population experience as a result of feeling competition and comparison from fellow students of the same age or graduate students in the same program. The interviewees indicated that they often face competition and comparisons from their peers, commonly known as involution. This pressure may stem from concerns about academic achievement, clinical practice, and employment prospects, leading graduate nursing students to constantly compare themselves to others in their pursuit of personal growth and progress, resulting in anxiety, restlessness, and frustration.

“We now have many students who have already sent out all their articles, passed their openings successfully, and met the conditions for graduation. However, our subject group has not yet started. Moreover, I see that other students seem to have started collecting data right away, but I have not started yet, and I feel a bit out of step” (N2).

“I probably feel pressured when I see my classmates around me publishing quality articles, and my supervisors want to see my research output and progress, and from time to time compare me with others, but I tend to make slow progress, which can cause me strong feelings of shame and unease” (N10).

“Feeling ungrounded, sometimes, I guess, thinking that looking at all the other students who are working so hard and still so good and still learning, am I going out and playing, how can I put it? Is it too indulgent? Playing is not fun, although not working, I always feel an invisible hand in control of me” (N3).

##### 3.1.1.2 Academic concealment and peer assault

A worrying trend is emerging in the academic environment of graduate nursing students: academic secrecy and zero communication with each other. Students often choose to conceal their research progress and personal learning skills, a practice that protects their own or their group’s privacy but also results in a lack of effective sharing of academic resources. Such a closed attitude not only hinders academic collaboration and innovation but can also breed mistrust and affect team cohesion. While it is vital to protect individual privacy, in the academic field, a moderate degree of openness and communication is the key to advancing knowledge.

“I did not submit one thing correctly for my article report at the time, and then the magazine called me back several times, and I was I even tried to add one of the students in my class because she had posted a similar article and wanted to ask for advice on how to change it, and I requested to add him as a friend, and he didn’t agree to it and rejected my request for help. This led me to think that I might do the same to others in the future” (N9).

“Everyone is covering up, and when you are helpless, your classmates just don’t share some of their stuff with you either. This is the time when you do not know whether your progress is among your classmates or not. On the top or middle or behind, just this feeling, I feel powerless” (N5).

At the same time, many subjects said that there is serious, vicious competition among nursing graduate students, and peer aggression occurs from time to time. Many of them will seriously exclude and suppress their competitors or even plagiarize the research results of others because of the competition for scientific research resources, scholarships, or other benefits. These behaviors not only hurt the rights and interests of the victims but also pollute the atmosphere of the whole academic environment.

“The competition between our classes is very fierce, especially when facing competition for resources and scholarship competitions. Some people will even gang up and maliciously suppress their rivals, engage in schoolyard bullying, or maliciously denigrate others, and this kind of learning environment has created a great psychological shadow on me. It has also seriously depleted my academic enthusiasm” (N14).

“Comparisons will exist between my fellow students and I. Sometimes, the other person will deliberately take the credit in front of the mentor or even report it maliciously. It feels like wasting a lot of time and energy on something meaningless and off the right track of scientific research, but it’s not that I want to do so, but I am forced to do so” (N8).

#### 3.1.2 Lack of learning resources

##### 3.1.2.1 Shortage of graduate nursing faculty

With the continuous development of the nursing discipline and the expansion of graduate student enrollment, there is an increasing demand for high-quality mentors. However, the reality is that there are problems such as an insufficient number of mentors, limited teaching experience, or Lack of scientific research ability of some mentors, which leads to uneven quality of postgraduate training. This not only affects the academic growth and career development of nursing graduate students but also restricts the overall progress of the nursing discipline.

“I think that the mentors, if you look at the mentors, are too uneven on the domestic side. A lot of clinical teachers have more work experience but not enough research ability. However, his title and seniority are up to par, and he can start enrolling students, which I actually don’t think is good. As graduate nursing students, we need teachers with research ability to lead us to learn and progress and provide us with research guidance and help” (N6).

“My mentor belongs to an external mentor; that is, he is on the administrative side inside the hospital. He is the bigger leader, but he just doesn’t have much energy to take me. When I saw other students following their mentors to learn various research methods, participate in research projects, and publish papers, I was under much pressure. So, I was in a state of confusion and helplessness almost most of the time” (N8).

“Only a small number of the teachers I know are in the nursing program because there are a limited number of study nursing teachers, and almost all of the other instructors are clinical, dental, con, or public health. Although they belong to the same medical category and the teachers’ abilities are outstanding, there is a generation gap because of the difference in specialties. Often there is no effective communication with us to give professional guidance and help” (N15).

#### 3.1.3 Difficulties in clinical internships

##### 3.1.3.1 Heavy internship tasks

Nursing graduate students often face the dual pressure of internship and research in their academic careers. On the one hand, they need to invest a lot of time in clinical practice, facing complex and changing patient situations and stressful working environments to improve their nursing skills continuously; on the other hand, the research task is also very difficult, requiring them to conduct in-depth research, data analysis, and thesis writing, which puts a very high demand on their academic ability and time management skills.

“I would feel very tired throughout the day after spending so much time in the clinic during the day and encountering a department with a high workload, then when I came back at night to do my research, I would often be tempted to go to sleep, or if I were in the library, it would be very difficult to settle down to study, and I found that other students around me felt the same way (N1).”

“Because of working and collecting data in the clinic and even helping the nursing faculty at the hospital with some of their research tasks (unpaid), right after I came back and went home, I was so tired, I did not want to do anything, and all I wanted to do when I got home was lie down. It is not that I subjectively do not want to do research work; I just cannot fight the physical fatigue (N3)”

##### 3.1.3.2 Difficulty in conducting scientific research

Nursing graduate students often face difficulties in conducting research in the clinical setting, mainly due to factors such as heavy clinical tasks, limited time and resources, and inadequate research conditions. These limitations limit their ability to explore nursing issues in depth, implement research programs, and produce high-quality research results.

“I feel that what is taught in school is very much not well represented and difficult to implement into actual clinical work, and many patients and even hospital staff do not support nursing research” (N6).

“In my case, because the teacher is inside the college and my internship hospital is not affiliated with the school, when I need to conduct ethical audits or collect data, I usually cannot find a channel or seek help, which leads to the slow progress of my project, and I am worried about graduation” (N9).

### 3.2 Coping mechanisms for negative experiences

Participants used a range of coping mechanisms, including internal regulation and external support, to adapt to and resolve the issues they faced and mitigate the negative impacts of their experiences.

#### 3.2.1 Internal regulation

##### 3.2.1.1 Psychological suggestion

Positive psychological hints can help nursing graduate students enhance their self-confidence and adjust their mindset so that they can better cope with the challenges encountered in research.

“At first, I was very anxious, and then after a period of adjustment, I kept cheering myself up, realizing that things still depend on myself to do and that only by going to put in action can it be somewhat effective, and I tell myself every day that you must be able to do it if others can, and that my efforts will definitely be rewarded” (N15).

“This process of scientific research is to produce the idea that internal conflict and anxiety are very normal, just like playing the game needs to upgrade and fight monsters. However, it is important to try to adjust your stress actively. And then it is to turn it into a kind of motivation and keep learning from experience, which is quite helpful for me” (N4).

##### 3.2.1.2 Diversion

When participants had accumulated a certain degree of negative emotions, they chose to escape temporarily, reducing their physical and mental burdens by engaging in enjoyable activities or doing what they wanted to do and temporarily forgetting about their stress.

“When I am very depressed, I will stop studying and working for a while and choose to go out with my good friends for dinner, travel, or exercise so that I can release my pressure. When I continue to come back to study after my state is adjusted, I find that I am more efficient” (N13).

“I like to buy myself something delicious, like dessert, or watch light-hearted and funny TV dramas to take my mind off my worries for a while. Then it’s time to sleep more; I feel much better when I wake up from sleep or go for a walk in the park, hiking, etc., or something like that to get some fresh air” (N6).

##### 3.2.1.3 Academic motivation

On the research path, participants said that if they received some positive feedback or saw the scientific output, they were greatly motivated to be more enthusiastic about their work.

“When the things I was anxious about getting progress like there’s been no small paper, and then finally one day you get this acceptance notice, and you think. It is okay, it is less anxious, and it even sparked my passion and interest in research” (N14).

“My mentor, family, and friends often comfort me and encourage me, especially when I finish a piece of work or make progress in my research, which makes me more motivated to do research” (N12).

#### 3.2.2 External support

##### 3.2.2.1 Guidance from seniors

On the path of nursing postgraduate students, the guidance of seniors is like a bright light to illuminate the way forward. They share their professional knowledge, clinical experience, and research methods so that we can take less detours and grow faster.

“My senior, who belongs to the category of seniors and has traveled the path we are on now, already has much experience in research, and he is willing to guide me, which is just very helpful to me” (N12).

“My senior sister is very willing to help me, no matter what, in study and life, she is willing to share a lot of knowledge and experience with me; we are also good friends; we will often go out to party, and there is no pressure to get along with her” (N5).

##### 3.2.2.2 Emotional compensation

Emotions are a bridge to establishing or maintaining connections with others. Participants mentioned that they shared their problems with family and friends, both online and offline, and that this method provided them with an outlet for emotional release and an important way for others to understand and support them.

“I would regularly call my family and friends who also cared about everything I was doing and got much energy from expressing my stress to them” (N11).

“I usually video call or meet up with the person I am in a relationship with, and I feel so much better and recharged after every meeting” (N8).

##### 3.2.2.3 Network empathy

Participants reported emotional resonance and interaction triggered by their common topics of study pressure, research challenges, and employment prospects. By sharing experiences and exchanging tips, they not only gained emotional support but also formed an online community of mutual support and cooperation.

“I often get some sense of identity from the Internet, that is, first I would go to brush up on Xiaohongshu, that is, to brush up on the kind of postgraduate students are not all facing a similar situation, and then it is just to confide in everyone and motivate each other” (N6).

“I’m more introverted and prefer to be alone, so I like to interact with others on social software and confide in each other; we don’t know each other or who the other person is, so we can say whatever we want” (N13).

### 3.3 Positive effects under dual pressure

The combination of internship and research work during this period undoubtedly created psychological and physical stress for the participants. However, these pressures provided them with a synergistic drive. Participants believed that their stress tolerance, research skills, and general qualities were enhanced and expanded through intense training under this dual stress.

#### 3.3.1 Tougher willpower

Nursing study is a profound exercise of willpower. Faced with heavy coursework, rigorous clinical practice, and the constant emergence of new knowledge and skill operations, participants need to be persistent and constantly challenge their limits. This process not only sharpened their professional skills but also forged the spirit of perseverance.

“Before grad school, I was in a state of laziness about everything, and when I encountered difficulties, my first reaction was to retreat and escape, but now I think more about how to solve problems and have higher expectations of myself” (N11).

“I find that I now have a higher level of concentration on my studies and work, and I try to learn new skills, knowledge, and keep breaking through the unknown” (N7).

#### 3.3.2 Greatly improved scientific research ability

Participants said that although the study and work were tough, they gained a lot of scientific research skills and methods compared to other interns, and their scientific research ability was also improved.

“Before, I did not know much about scientific research and these things. I did not even know what scientific research was, but now, if I’ve learned a little bit, I guess, in general. And now I know how to identify research problems in practice and how to use research to promote clinical progress” (N9).

“I do not care if you have a paper or not, or if you have a good journal, but I do not know how to write before, but now I can at least write, right?” (N11).

#### 3.3.3 Improvement of comprehensive quality

Through systematic learning and clinical practice, their comprehensive quality is significantly improved. Not only has professional knowledge been deepened, but clinical skills are also more skilled, and interpersonal skills, communication, and coordination skills have been cultivated.

“The internship work is for us personal learning growth or some other aspects, or it has a certain promotion and positive effect. I also met many excellent teachers who taught me how to communicate with patients, and I gained a lot” (N6).

“Through the problems identified in the clinical placement, so much so that it means that the research that we are allowed to do will not be so vague, I am also becoming more and more aware of how important it is for nurses to be responsible and empathetic. It takes a lot of time and dedication to do something well.”

### 3.4 Inner needs

After the accumulation of internship and research work, participants gradually have a clearer understanding of themselves and their surroundings, and better understand their different needs when facing the dual pressure of internship and research.

#### 3.4.1 Adequate research resources

Numerous participants indicated that they faced a serious lack of research resources, which included faculty, study materials, research skills, data collection, and some financial subsidies for internships.

“As far as I understand, many students around me, they are actually on their own in that feeling of crossing the river by touching the stones, that is, actually are all relying on themselves to learn, and very much hope that there will be someone to lead you on top of the scientific research, including a mentor, preferably related to the nursing specialty, and a mentor from the school and the internship hospital respectively, to co-manage on top of the scientific research as well as the clinical time” (N1).

“What I think is that there are some courses, is it possible to replace them with more courses on research, and provide some opportunities to attend academic conferences, and there are few databases in our school, and we often need to pay for them ourselves” (N4).

“It will be difficult to find research subjects for my research, I hope the practice site can provide some ways to collect questionnaires or conduct experiments” (N6).

#### 3.4.2 Flexible internship management model

The participatory system indicated that they needed different management and teaching styles from undergraduate students, especially during the placement phase, which would be more conducive to learning and working.

“I am a nursing specialist, and then I am going to be in a clinical placement for 18 months. Moreover, I have looked online at some of the nursing postgraduates from other schools, but it seems like some of them only work half-day shifts, so they can leave some of their time to study and improve their research skills” (N2).

“I would like the leave of absence process to be less cumbersome during my clinical practicum when I need to go collect data for my research or when I have to go back to school or to my mentor for something, as it is often an emergency notification” (N13).

“I would like to minimize the workload of graduate students, that is, to stop working as much as undergraduates, and I hope to have more interaction with clinical faculty so that research and clinical integration can take place.”

Graduate nursing students are often under financial pressure during their practicum phase, lacking a stable income because they are not formally employed. Providing a certain level of financial support is important to ensure their quality of life and facilitate the successful completion of their studies.

#### 3.4.3 Financial assistance

Nursing graduate students often face financial pressure during the internship stage and lack stable income because they are not formally employed. Providing some financial support is important to ensure their quality of life and promote the successful completion of their studies.

“I need to rent my own room for my internship, some hospitals provide free accommodation, and then the dormitory environment is also quite good, it’s a dual room, but it’s not like my side, I also have to just have to pay my own rent and utilities every month, and it’s also quite expensive to eat take-out” (N12).

“For graduate students who are in their twenties, they still have to reach out and ask their parents for money, which is actually quite embarrassing, and I think that the internship treatment can be improved a little bit” (N7).

## 4 Discussion

The psychological experience of master’s degree nursing students presents complex and multidimensional characteristics when they face the dual pressures of clinical practice and research tasks at the same time. The dual challenges of internship and research work appeared to be a significant source of negative emotions among graduate nursing students in this study (19–21). Exploring the experiences and feelings of nursing students under “dual pressure” and their coping mechanisms in the face of adverse experiences is of great significance to better maintain their physical and mental health and improve clinical nursing education. The combination of clinical practice and research is also conducive to the development of their time management skills, stress tolerance, and the enhancement of their professionalism. However, it is worth noting that as they progress, their stress level increases accordingly. Excessive stress may reduce nursing students’ self-confidence, enthusiasm for research, and professional interest (22–24). In our study, the dual challenges of internship and research work were the main causes of negative emotions among graduate nursing students. The heavy clinical internship tasks and daily trivial affairs occupied a lot of time and energy, which led to fatigue of their organism, making it difficult for them to fully devote themselves to scientific research, and even burnout of scientific research.

Peer aggression and peer pressure further aggravate the psychological burden (25, 26). In a competitive academic environment, some students may use improper means, such as malicious reporting and defamation, in pursuit of short-term results or awards and prizes, which not only undermines the academic culture but also causes psychological harm to other students (27, 28). Most of the participants reported that peer pressure made them prone to negative emotions such as anxiety and self-doubt when confronted with the scientific achievements of others, which further weakened research motivation (29). The phenomenon of academic concealment is also more prominent, and students often choose to keep their research progress closed for fear that their research results will be published first or their resources will be taken away, which hinders academic communication and cooperation (30). These psychological experiences reflect the deep-rooted problems in the training system of nursing master’s degree students. Fewer nursing faculty and insufficient research resources are the main external factors (31). The limited number of mentors and the relatively small percentage of nursing faculty mentors make it difficult to provide professional and research guidance to each student. In contrast, the imbalance of research resources restricts the depth and breadth of students’ research, which makes it difficult for them to make progress on the research path (32).

Research has shown that “talking” is the most common coping method among nursing students. By talking to friends, family, or classmates, participants could relax and forget about the problems they faced during their internships and studies for a while, thus alleviating some of their negative feelings and better facing their current challenges (33). Mental cues and seeking external support are positive responses to negative experiences (34). Confidence can be greatly increased by establishing a positive psychological state, while internal regulation is a key measure for dealing with internship and research conflicts. Previous research has shown that. Understanding the stress-coping strategies of graduate nursing students can help create a caring and supportive learning environment that further assists graduate nursing students in achieving nursing success (35). Faced with stress, different students exhibit completely different coping strategies. According to Lazarus and Folkman’s theory of stress and coping, the core reason for this difference lies in the dynamic interaction between cognitive appraisal and coping resources. Firstly, in the primary appraisal stage, the former may view dual pressures as a “challenge” to prove their abilities, while the latter is more likely to see them as a “threat” to self-esteem. Secondly, in the secondary appraisal, the former believes they have sufficient social support and time management skills to handle difficulties; the latter feels a lack of control due to insufficient resources. This difference leads the former to adopt problem-focused coping centered on solving issues, creating a positive cycle in action, whereas the latter, driven by helplessness, engages in emotion-focused coping and may even displace anger onto peers to vent frustration and maintain a false sense of self-worth. Nursing master’s degree students are the backbone of future nursing development, and paying attention to their mental health and optimizing the training system is important for improving nursing talent, training quality, and promoting the development of the nursing discipline. Nursing administrators and educators need to pay great attention to the psychological state of graduate nursing students in the “dual stress period,” actively identify problems, and provide necessary help and support to help them get through the difficulties.

## 5 Strengths and limitations

### 5.1 Strengths

Online interviews overcome the physical distance between researchers and interviewees. Interviewees can choose the time and place of their interviews, which makes them more relaxed in a familiar environment. Thus, online interviews improve the reliability and authenticity of the research data. In addition, the nursing students interviewed came from hospitals and schools in different provinces in China, including the east, west, south, and north. The study’s scope was wide, so the results were more representative.

### 5.2 Limitations

This study had several limitations. First, although participants were recruited from 11 universities across China, the sample was predominantly female (12 out of 15), which reflected the gender imbalance in nursing but limited the transferability of findings to male graduate nursing students. Second, the study relied solely on online interviews. While this approach overcame geographical barriers, it prevented the researchers from fully capturing certain non-verbal cues that might have been observable in face-to-face settings. Third, all data were self-reported, introducing the possibility of recall bias and social desirability bias. Participants might have underreported negative emotions despite the researchers’ neutral stance. Fourth, the cross-sectional design captured experiences at a single time point, so the findings did not reflect how these experiences might evolve over time.

## 6 Conclusion

Four main themes were extracted from this study. The professionalism, general competence, and adaptability of graduate nursing students were enhanced during the internship and research process. However, the negative experiences encountered by nursing students are an area that hospitals and schools should pay more attention to. It is suggested that schools and hospitals should strengthen contact and communication, provide more humanistic care, and meet all the needs of their dual-stress period as much as possible. In addition, adequate learning resources and research platforms should be provided, and the nursing faculty should be strengthened to ensure students’ basic research needs are met. It is vital to establish a favorable academic environment and strengthen moral and ethical development, which will help to improve the overall quality of care and increase their professional identity, which confidently faces the challenges of internship and learning. The training of nursing master’s degree students should pay more attention to the overall development of the students, which should not only improve their clinical practice and research ability but also cultivate their teamwork spirit and diversify the evaluation system. We hope that by optimizing the training system, strengthening the resources, and improving the evaluation mechanism, nursing master’s degree students will be able to find a balance under the dual pressure of clinical and scientific research and grow into high-level nursing talents with both practical ability and scientific research literacy.

## Data Availability

The original contributions presented in the study are included in the article/supplementary material, further inquiries can be directed to the corresponding authors.
